# Advances in electrospun nanofiber-based personal protective equipment for active filtration

**DOI:** 10.1186/s11671-026-04693-5

**Published:** 2026-06-02

**Authors:** João S. Oliveira, Sofia M. Costa, Joana C. Araújo, Raul Fangueiro, Diana P. Ferreira

**Affiliations:** 1 Centre for Textile Science and Technology (2C2T), University of Minho, 4800 Guimarães, Portugal; 2 Department of Textile Engineering, University of Minho, Guimarães, Portugal

**Keywords:** Facemask, Biopolymers, Natural fibers, Biodegradable, Electrospinning

## Abstract

The rapid increase in air pollution and airborne pathogen transmission has driven the demand for advanced personal protective equipment (PPE) that combines high filtration efficiency whit environmental sustainability. Conventional facemasks are primarily composed of non-biodegradable synthetic polymers, resulting in microplastics pollution and long-term ecological damage. Electrospinning offers a promising approach for producing nanofiber membranes with ultrafine diameters, high porosity, and tunable surface properties, enabling efficient particle capture and antimicrobial functionalities. Biodegradable polymers present sustainable options, as they can form electrospun membranes with high porosity and interconnected structures that effectively capture airborne particles while reducing environmental impact. These materials can also be enhanced with bioactive agents, including metal nanoparticles (NPs), metal–organic frameworks, and essential oils to provide antibacterial properties. In addition, naturally derived fibers are explored for use in the outer and inner layers of facemasks, offering breathability, comfort, and biodegradability. Together, these materials pave the way toward fully bio-based facemask architectures that combine high filtration efficiency, bioactivity, and sustainability. This review explores the potential of biopolymer-based electrospun nanofiber membranes and natural fibers for use in biodegradable facemasks, with a focus on their filtration efficiency, antimicrobial functionality, and overall suitability as a sustainable solution for future PPE applications.

## Introduction

Air pollution is a major global health concern, contributing to a significant number of deaths worldwide through its association with pulmonary disorders, cardiovascular events, and various types of cancer [1]. Air pollution can be defined as a complex mixture of components referred to as particulate matter (PM). This mixture encompasses toxic gases, such as carbon monoxide, carbon dioxide, volatile organic compounds (VOCs), and nitrogen oxides; biological pollutants, including spores, bacteria, and viruses; as well as solid and organic particles, such as sulfates, nitrates, ammonia, black carbon, mineral dust, aerosol particles, benzene, polycyclic aromatic hydrocarbons, and water [1, 2]. PM is commonly classified into three categories: PM_10_, PM_2.5_, and PM_0.1_. Particles with diameters smaller than 10 µm are classified as PM_10_, which are partially filtered by the nasal passages. Particles with diameters bellow 2.5 µm (PM_2.5_) and 0.1 µm (PM_0.1_) are defined as fine and ultrafine particles, respectively [3]. Unlike larger particles, fine and ultrafine particles can bypass the filtration mechanisms of the upper respiratory tract, infiltrate into the pulmonary system, and accumulate in the alveoli, where they may induce both direct and indirect effects on the cardiovascular system [4].

An effective strategy to protect the human being against PM and other environmental hazards is the use of personal protective equipment (PPE). The proper use of PPE is intended to prevent the entry of PM into the respiratory and cardiovascular systems, thereby reducing the risk of adverse health effects. PPE includes a wide range of items, such as gloves, goggles, gowns, and facemasks, with facemasks representing the most widely used form [5]. These disposable, loose-fitting devices are designed to cover the mouth and nose, serving as a barrier against potential pollutants, including PM and biological pathogens [6].

The unprecedented global spread of coronavirus disease 2019 (COVID-19), caused by severe acute respiratory syndrome coronavirus 2 (SARS-CoV-2), emerged as a major public health concern in 2020, with the potential to cause severe pneumonia, acute myocardial injury, and long-term cardiovascular damage [5, 6]. Aiming to reduce transmission of this virus, several preventive measures were strongly recommended, including frequent hand hygiene, social distancing, avoidance of crowded places, and the widespread use of facemasks [5, 7, 8]. This surge in preventive measures resulted in an exponential increase in the global demand for surgical facemasks, with the World Health Organization (WHO) estimating a monthly requirement of 89 million units worldwide in 2020 [9]. For instance, in that year, China increased its daily output to approximately 14.8 million facemasks, while Japan reported a monthly production of around 600 million units [10]. The large-scale production and improper disposal of disposable facemasks, which are predominantly composed of non-biodegradable, petroleum-derived polymers such as polypropylene, polyethylene, polyurethane, polystyrene, polycarbonate, and polyacrylonitrile, have raised significant environmental concerns, as they contribute to the growing accumulation of microplastic [11–13].

This highlights the urgent need to develop novel biodegradable facemasks that are fully bio-based, efficient, affordable, and comfortable, while simultaneously reducing environmental impact [13]. Nonetheless, it is crucial to further develop facemasks that not only provide effective filtration but also actively inactivate or degrade microorganisms. Although conventional facemasks can efficiently filter airborne particles, the risk of microbial contamination on their surfaces remains high after prolonged use [14]. Moreover, the disposal of used facemasks poses a significant risk of secondary contamination, as numerous microorganisms and viruses can remain on their surfaces [15]. Functionalizing facemasks with antimicrobial agents can significantly enhance their protective performance by actively neutralizing potential contaminants. Incorporating such agents represents an important advancement in improving the overall efficacy of facemasks for wearer protection [14–16].

The classification and functionality of polymer membranes are closely linked to their pore size, which defines their suitability for different separation processes: microfiltration membranes (0.1–5 μm) can retain bacteria and protozoa; ultrafiltration membranes (0.01–0.1 μm) target proteins, viruses, and colloids; nanofiltration membranes (1–10 nm) remove small organic molecules and divalent ions; and reverse osmosis membranes (0.1–1 nm) provide the finest separation, making them highly effective for desalination and the removal of very small solutes [17]. Several fabrication methods, including phase inversion, interfacial polymerization, and casting, have been employed to produce porous membranes; however, electrospinning stands out as the most widely adopted technique, owing to its simplicity, cost-effectiveness, and ability to generate micro/nanofiber membranes with controllable fiber diameters and pore structures [18].

Electrospinning technology has recently attracted considerable attention for the fabrication of high-performance polymer membranes, particularly for filtration applications. By applying high voltage to overcome the surface tension of a polymeric solution, electrospinning enables the formation of continuous fibrous structures with diameters ranging from the nano (< 100 nm) to microscale (> 1 µm), with complete solvent evaporation during the process [19]. The resulting electrospun nanofibers possess several advantageous characteristics, including a high surface-area-to-volume ratio, tunable morphology, interconnected porous networks with small pore sizes, and adjustable mechanical performance [20]. Beyond filtration, electrospinning has also emerged as a promising approach for the encapsulation and surface functionalization with bioactive compounds - both hydrophobic and hydrophilic - as well as larger macromolecules, thereby enhancing the bioactivity, biocompatibility and bioaccessibility of the fibers. Consequently, electrospun membranes are being investigated for diverse applications, including wound dressings, drug delivery, tissue engineering, and protective clothing [21].

Significant research efforts are currently focused on enhancing the performance of facemasks by developing and selecting advanced materials that provide antimicrobial activity, high filtration efficiency, reusability, low weight, comfort, and environmental sustainability. In this context, functionalized electrospun fiber-based facemasks have emerged as a promising solution due to their tunable properties and multifunctional capabilities. However, a comprehensive overview of recent advances in this field is still needed to guide ongoing and future research. Therefore, this review aims to provide an understanding of the structure and function of facemasks, summarize current developments in electrospun facemasks with antimicrobial and biodegradable characteristics, and discuss emerging opportunities for the advancement of next-generation facemask technologies.

## Facemasks

### Function

The main function of a facemask is to protect the user from environmental hazards present in air. Environmental hazards from polluted air include various types of toxic gaseous chemicals such as sulfur oxides, nitrogen oxides, carbon oxides, formaldehyde and a combination of other VOCs [22, 23]. Asthma, anemia, and nervous system effects may result from interactions between high concentrations of VOC exposure and the human body [21]. Additionally, bioaerosols including bacteria, fungi and viruses represent another category of pollutants that significantly increases the hazard to public health due to their ease of rapid transmission through polluted air [24, 25]. These biological pollutants have the potential to produce allergic, infectious, and toxic illnesses, both chronic and acute [26]. On the basis of the preceding analysis, concerns about airborne contaminants, such as PM of various sizes, chemical mixes, and microbiological particles, have grown when defining air quality, particularly indoor air quality [24, 26]. PM is a trace air pollutant that has been identified as a significant risk factor for premature death [27]. As mentioned above, airborne particulate pollution is classified into ultrafine particles (PM_0.1_), fine (PM_2.5_) and coarse (PM_10_) particles. When PM concentrations rise, the resulting pollution is the primary cause of human disease [28–30]. PM_10_, also referred to as coarse particles (diameters < 10 µm), primarily originate from crustal material suspensions, including dust, soil, agricultural activities, mining, and residues from natural disasters, as well as from sea salts, spores, pollen, and mold [31]. These particles are generally deposited in the upper respiratory tract, where they may be blocked by the nasal passages or deposited in the trachea and bronchi [32, 33], and thus are considered less hazardous compared to fine and ultrafine particulate matter. PM_2.5_ (fine) and PM_0.1_ (ultrafine) refer to airborne particles with smaller size, high penetration rate, and that can easily carry a variety of toxic substances that can cause cell death or organ dysfunction, both of which are extremely dangerous [34–37]. These particles enter the bronchi directly through the nasal cavity and connects to the lungs, causing not only respiratory problems such as bronchitis, lung cancer, asthma [38, 39], but also cause kidney impairment and, in severe cases, kidney failure and a high risk of abnormalities in pregnancies [40–42].

The suitability of different mask types is determined by several factors, including working conditions, type of illness, or other medical requirements. For instance, surgeons and mineworkers need to use different types of masks derived from the different working conditions. Facemasks are commonly divided into two types: surgical masks and respiratory masks. The main function of surgical masks is to protect the patients by preventing droplets, bacteria and aerosols not smaller than 3 µm from the user’s nose or mouth, while respiratory masks allow the filtration of smaller particles ranging from 0.01 to 0.3 µm with a filtration performance superior to 95%. These facemasks are generally used in hazardous/toxic atmospheres [11, 21].

**Fig. 1:**
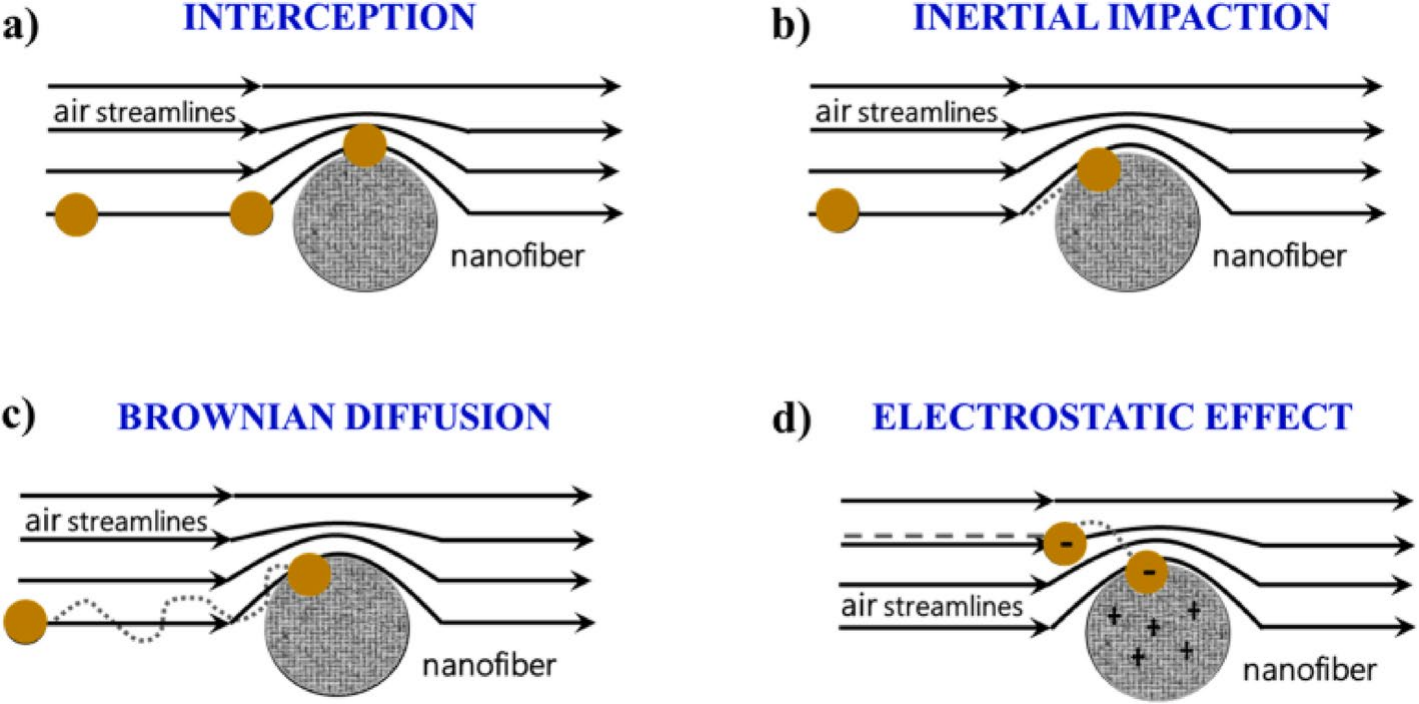
Schematic representation of the primary filtration mechanisms in facemasks: **a** interception, **b** impaction, **c** diffusion, and **d** electrostatic attraction. Retrieved from [3]

Filtration efficiency can be categorized into two distinct modes: steady-state and non-steady-state. In steady-state conditions, filtration performance is predominantly regulated by the intrinsic properties of the filter material, airflow velocity, and particle characteristics. In contrast, non-steady-state conditions account for the effects of filtration duration and particle buildup on the filter [43]. Facemasks are capable of filtering different particle sizes (macro, micro and nano) through different types of mechanisms: interception, impaction/collision, diffusion and electrostatic attraction (Fig. 1). The interception mechanism is effective in macro particles, as they have larger sizes than the pores of the fibers, being immediately intercepted while contacting nanofiber surface due to van der Waals forces. In the case of the impact/collision mechanism, micro-scale particles are able to move between the pores but can collide with the fiber walls. Nanoscale particles, due to their small sizes, can easily move between the pores without colliding with the walls, and the impact/collision mechanism is not very effective in this case. With this, the diffusion mechanism is efficient in filtering nanoscale particles. Diffusion occurs when the random (Brownian) motion of a particle causes that particle to contact a fiber. As a particle vacates an area within the media, by attraction and capture, it creates an area of lower concentration within the media to which another particle diffuses, only to be captured itself [44]. However, sometimes micro/nano particles do not respect these mechanisms, leading to the use of several layers of fibers, raising some problems in terms of the breathability of the masks. In an electrostatic attraction mechanism, filtration takes place through fibers charged with opposite charges to the particles, thus being able to attract the particles [45, 46].

Facemasks are thus expected to serve a dual function: protecting the wearer from environmental contaminants such as dust, bacteria, and respiratory droplets, while allowing for adequate air permeability and playing a critical role in containing the expulsion of respiratory emissions, such as those generated by coughing or sneezing, by the wearer, thereby helping to reduce the risk of transmission and maintain a safer surrounding environment [21].

### Structure and composition

**Fig. 2:**
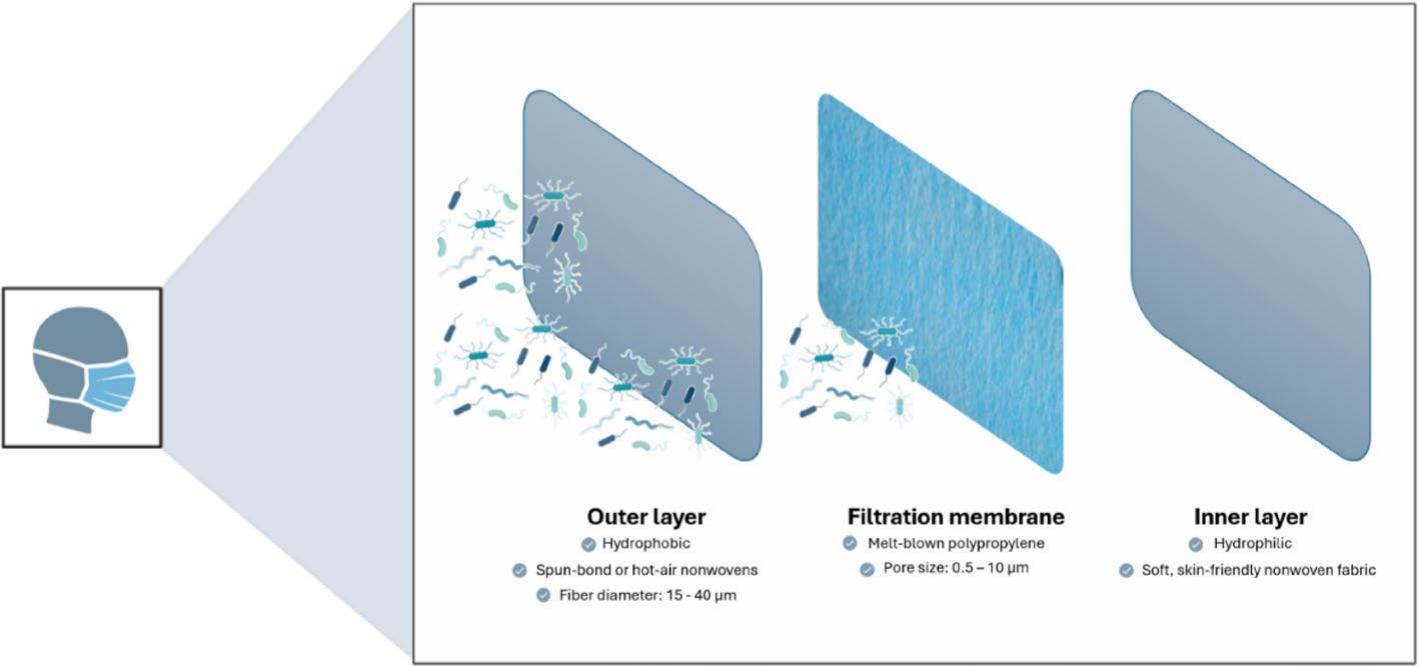
Schematic representation of the composition of a traditional disposable facemask, consisting of outer and inner layers made of nonwoven fibers and a melt-blown polypropylene membrane serving as the primary filtering layer

A typical facemask consists of a multilayered composite structure, commonly comprising three distinct layers (Fig. 2): an inner layer (soft, skin-friendly nonwoven fibers); a middle layer (melt-blown filter, which functions as the primary filtration barrier); and an outer layer (nonwoven fibers that is water-resistant and often colored) [12, 21]. For the adsorption of VOC, an additional middle layer of activated carbon fabric may be added [47].

The inner layer should be soft nonwoven fabric, as it is in direct contact with the skin, providing comfort to the user, and hydrophilic, to hold cough droplets. The outer layer is often hydrophobic and has the function of preventing the passage of liquids as well as blocking the entry of larger particles. These layers are usually spun-bond nonwovens or hot-air nonwovens with a fiber diameter of 15–40 µm. They do not possess great impact on total filtering effectiveness, but they are resistant and have proper air permeability. A performance filter membrane, often made of melt-blown polypropylene, with small pores (0.5–10 µm) and excellent filtration efficiency makes up the middle layer of the membrane [19, 21]. Filtration media is usually produced by techniques such as melt-blowing, spunbonding and electrospinning. Melt-blowing is a technique where a molten polymer is extruded through a fine die into a high-velocity hot air jet, which stretches and cools the polymer, causing it to solidify and deposit onto a moving collector, where fibers bond to form a nonwoven web [48]. In the spunbonding process, molten polymer is extruded through multiple nozzles to form continuous filaments, which enter a duct where they are cooled by quench air and subsequently drawn by suction and pressurized air. The filaments are then bonded by thermal, chemical, or mechanical methods to form a nonwoven fabric [49, 50]. These techniques produce nonwoven fabrics with fiber diameters in the micrometer range, typically between 1 and 50 µm for spunbonded materials and up to approximately 7 µm for melt-blown fibers [50, 51]. Filter media produced using both techniques can efficiently remove PM in the micrometer size range. However, particles with nanometer-scale diameters remain challenging to capture, as they do not conform to a single filtration mechanism. Consequently, multilayer filters are required, resulting in reduced breathability and increased pressure drop [51].

Therefore, electrospinning - a process in which a polymeric solution is stretched under an applied electric field to produce continuous nanofibers - represents a promising alternative. Electrospun nanofiber membranes exhibit reduced pore sizes due to their uniform fiber diameters, typically ranging from 50 to 100 nm, as well as high specific surface area and enhanced surface adhesion. These characteristics promote increased particle capture, resulting in high filtration efficiency while maintaining low airflow resistance [21].

In addition to polypropylene, other non-biodegradable polymers are also used for the production of the three layers of surgical masks, such as polystyrene, polyethylene, polyamide, polyester and polycarbonate [43, 52]. Facemasks are then disposed of into dumps and landfills, and some are simply littered in public, and unfortunately, they end up in coastal, marine, and terrestrial environments, resulting in massive amounts of polymeric waste. They can fragment into smaller size/pieces of particles under 5 mm, emerging as a new source of microplastic fibers. The global increase in the production and consumption of facemasks due to COVID-19 pandemic exacerbates the environmental risk associated with improper disposal. According to the results of an experiment conducted by Saliu et al*.*, a single surgical mask can release up to 173 000 fibers into the environment per day [12, 53, 54]. Although facemasks provide protection against droplets and splashes, they exhibit several limitations, including low efficiency in filtering fine particles, reliance on non-biodegradable polymers, and lack of reusability [55]. These shortcomings highlight the need for innovative strategies to design facemasks with enhanced filtration performance, sustainable material choices, scalability in production, and the integration of bioactive functionalities. Electrospinning represents a promising technology to address these challenges, owing to its ability to produce nanofiber membranes with the above-mentioned characteristics.

## Electrospinning

With the development of nanotechnology in the last decade of the twentieth century, techniques such as electrospinning gained worldwide attention in the production of polymeric fibers at the micro and nano scale [56]. Over the last few years, this technique has been attracting attention from both the scientific community and industry due to its great potential for responding to all major global challenges [57]. The versatility of electrospinning, along with its simplicity of use, accuracy, cost-effectiveness and scalability, make this technique very attractive for different applications, namely for the development of wound healing devices, food packaging, drug delivery systems, tissue engineering, among others, including for air filter systems [19, 57–59].

**Fig. 3:**
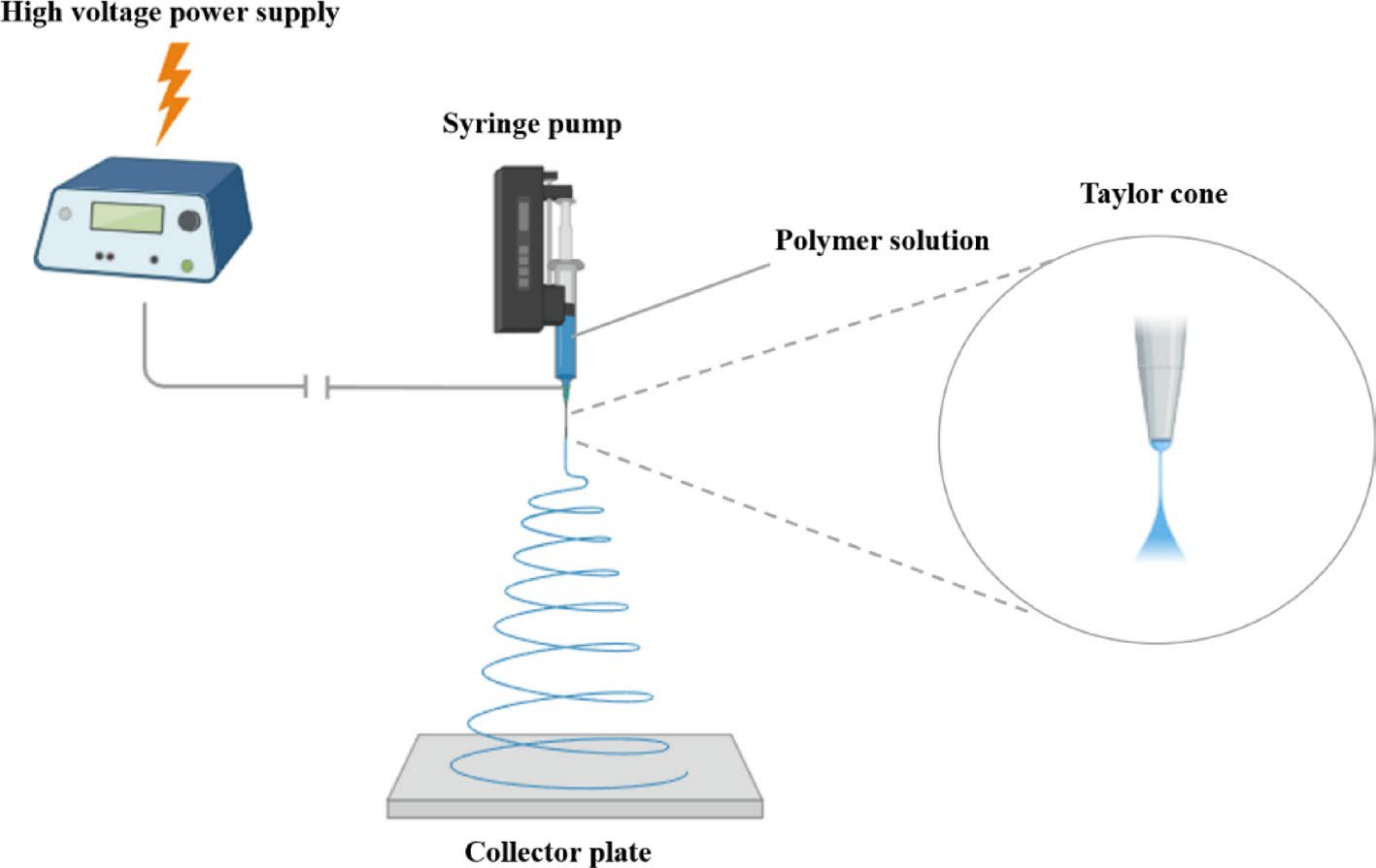
Schematic representation of the electrospinning setup. Created with BioRender.com

Electrospinning is a process to manufacture nanofiber materials with the assistance of a high electrical voltage applied to polymer solution (Fig. 3) [21]. To establish a charge imbalance, an electric field is supplied to the polymer melt. The introduction of the electric charge causes tension in the melt, which causes the charged jet to form at the cone's tip (Taylor cone). The liquid polymer solution is prepared for jet development after charging. The polymer solution swiftly elongates, the solvent evaporates, and forms nanofibers when the charged jet is immediately focused on the target. Thus, the target is fulfilled with solid nanofibers [60, 61]. Therefore, it is possible to conceptualize the electrospinning process as a procedure that effectively converts the polymer beads into polymer fiber products [21].

This technology is highly versatile as it enables the production of continuous nanofibers from a variety of polymers and is more user-friendly and affordable than alternative nanofibers processing methods. Different benefits of this technique include the ability to produce large quantities, easy functionalization of fibers, material combination, and deposition on other substrates [60]. The fibers produced by the electrospinning process can take on a variety of shapes, including yarn, membranes, and even three-dimensional scaffolds [62–64]. It is widely employed in many different applications, most notably in the biomedical field for the processing of tissue fibers at the nanoscale [60]. Nanofiber as a material for air filtration membranes has increasingly gained researchers attention, especially due to the combination of its large specific surface area and ultra-fine porosity [65].

An electrospinning device is made up of three major components: a receiving device, a jet bubbler, and a power supply. By changing the components of the electrospinning equipment, different types of sustainable materials can be produced. Some fibers, for example, can be spun onto a static collector, while others can be spun under a rotating drum. Furthermore, single-strand fiber can be spun with a single-axis needle, while multiaxis needles can spin multicomponent fibers simultaneously, and core–shell fiber can be produced with coaxial spinning. The electrospinning setup's electrical power supply can supply both alternating current (AC) and direct current (DC) [66]. When using a rotating drum collector, the metallic collector's movement and form can be altered in order to control the fiber orientation. To create randomly oriented fibers, a flat plate is employed; parallel plates/strips with clearly defined gaps and regulated electrostatic interactions help align nanofibers into a parallel array. Various kinds of rotating drums may produce distinct edge morphologies and collect geometries. The ability to run continuously provides the opportunity to scale up the manufacturing of nanofibers [67].

This technology is well established, affordable, simple to scale up, and approved by industries [68]. The electrospun filter media can provide great filtration efficiency due to the high porosity (≈80%), tortuosity, and large specific surface area [69]. Along with this, the electrospinning device can process a variety of polymeric materials, which is a necessity for biodegradable polymers to prepare the membrane media [67]. To create biodegradable nanomembranes for nano-filtration, a variety of biopolymers have been used, including alginate, chitin, chitosan (CS), polyvinyl alcohol (PVA), polylactic acid (PLA), polyethylene glycol (PEG), cellulose acetate, and poly(Ɛ-caprolactone) (PCL), as well as their blends [70]. In addition, these membranes can be functionalized with bioactive molecules to impart antimicrobial functionalities. Such functionalization enables the inactivation of captured microorganisms directly on the membrane surface, thereby reducing microbial survivability and mitigating the risk of re-aerosolization [71, 72].

### Electrospinning parameters

Several factors have to be considered in the electrospinning process along with the polymer characteristics, since it directly affects the morphology and fiber diameters [21, 60]. These factors influencing the electrospinning process are generally classified into three categories: solution parameters (solvent and polymer type and concentrations, viscosity, and electrical conductivity of the solution), electrospinning parameters (needle diameter, solution flow rate, distance between the needle and the collector, applied electric field strength, and the type of collector used), and environmental parameters (ambient temperature and relative humidity) [73].

#### Solution parameters

The electrospinning process relies on the uniaxial stretching of a charged polymer jet. Variations in the concentration of the polymer solution significantly influence the extent of jet elongation, thereby affecting the morphology and uniformity of the resulting nanofibers. The surface tension and applied electric field cause the entangled polymer chains to disintegrate before reaching the collector when the concentration of the polymeric solution is low [74, 75]. Insufficient polymer concentration in the electrospinning solution often results in the formation of beads or beaded nanofibers. As the polymer concentration increases, the viscosity of the solution also rises, promoting greater chain entanglement among polymer molecules. These chain entanglements help to overcome surface tension forces, facilitating the formation of uniform, bead-free nanofibers during electrospinning [73]. Solution conductivity has high influence on the formation of the Taylor cone and the resulting nanofiber diameter during electrospinning. In solutions with low conductivity, the droplet surface lacks sufficient charge to initiate Taylor cone formation, thereby preventing electrospinning. As conductivity increases to a critical threshold, the surface charge becomes adequate to form a stable Taylor cone, leading to the successful ejection of the jet and a reduction in fiber diameter due to enhanced stretching forces. However, increases beyond this optimal conductivity can destabilize the process and, therefore, inhibiting Taylor cone formation and disrupting electrospinning process [73].

#### Electrospinning parameters

It is generally accepted that at a critical voltage, a spherical droplet will deform into a Taylor cone and produce ultrafine nanofibers when current is introduced into a solution via a metallic needle [76]. Each polymer has a different critical value of applied voltage. The stretching of the polymer solution in combination with the charge repulsion within the polymer jet is thought to be the cause of the production of smaller-diameter nanofibers with an increase in the applied voltage [77]. Beads or beaded nanofibers will form if the applied voltage is increased beyond a critical point [73]. A crucial flow rate for a polymeric solution could be used to prepare uniform bead-free electrospun nanofibers. The polymer system affects this key value. If the flow rate is raised above the critical point, beads, ribbon-like flaws, and unspun droplets may develop. A minimal flow rate is preferred to maintain a balance between the departing polymeric solution and replacement of that solution with a new one during jet development. Increases and decreases in flow rate have an impact on the formation and diameter of nanofibers [73, 78]. The distance between the metallic needle tip and the collector must be optimized based on the specific polymer system, as it significantly influences nanofiber morphology, affecting solvent evaporation rate, deposition time, and the extent of the whipping or instability region during electrospinning [79]. The choice of collector also significantly influences fiber morphology. The use of a stationary flat collector typically results in randomly oriented nanofibers, whereas a rotating cylindrical drum promotes fiber alignment. This configuration generates membranes with enhanced tensile properties, which are desirable for a wide range of applications [80].

#### Environmental parameters

Humidity influences nanofiber diameter by regulating the solidification dynamics of the charged jet during electrospinning. However, the extent of this effect is dependent on the chemical nature of the polymer, as different polymers exhibit varying affinities for moisture and solvent evaporation rates [81]. An increase of humidity during electrospinning leads to the formation of small circular pores on the fiber surface. These are produced as a result of the solvent's evaporative cooling as it travels from the needle tip to the collector during electrospinning [82]. Additionally, at low humidity, the solvent is removed from the needle tip more slowly than it evaporates, clogging of the needle. This ultimately disrupts and may terminate the electrospinning process [83]. In terms of temperature effects, higher ambient temperatures will result in higher solvent evaporation rates, which will cause the charged jet to solidify more quickly. Also, the polymer solution's viscosity lowers, and because of this, fibers stretch more quickly, leading to the production of thinner fibers [84]. Furthermore, biological molecules like proteins and enzymes may lose their functioning at greater temperatures if they are employed in electrospinning [83].

## Potential of electrospun nanofiber membranes for air filtration applications

The study of electrospun technology's use in air filtration has become more in-depth in recent years. Electrospun nanofibrous mats have been recognized as powerful candidates for high-performance filters, since they present morphological advantages over current conventional filters [85], including higher water/air flow rates due to high porosity and small pore sizes [21, 86], ample space for functionalization of membranes to effectively remove multiple pollutants, including particles, bacteria/virus, and harmful gas [87], low air resistance for air filtration [88], and high surface-to-volume ratio [89]. Compared with conventional micro-fibrous filters, the nanofibers produced by electrospinning can increase the possibility of PM deposition [19]. By making nanofibers a suitable active material for many applications in the sectors of electronics, biology, energy storage, healthcare, and fabric technology, those properties have aided in the development of nanofiber technology [90].

Electrospun fibrous membranes represent a high-performance filtration platform. Air filters produced via electrospinning typically exhibit superior filtration efficiency compared to conventional filter media, primarily due to their unique structural features, including ultrafine fiber diameters and high specific surface area, resulting in enhanced particle capture while maintaining relatively low airflow resistance [86]. Many polymers are now employed to make electrospun materials for air filtration, and new single and multi-polymer filtration materials are continually being made [91, 92].

In current research on electrospun air-filter membranes, a single polymer is most commonly used to produce fiber membranes for PM filtration. The performance of such electrospun membranes depends on the properties of the selected polymer. However, single-polymer filter membranes typically serve a single function and often lack the versatility required to address the challenges posed by complex or multi-functional conditions [91, 93]. A variety of polymer materials can work together to address these challenges, aiming to meet the demands of more complex application environments [94]. The need for air-filtration materials has continually grown as a result of the environment's increasing complexity, and materials that satisfy a variety of property criteria are constantly being researched and manufactured. There are numerous approaches to manufacture materials for air-filters via electrospinning of different polymers, such as mixing, multilayer electrospinning, and multifluid electrospinning [91].

The possibility to tailor nanofibers’ composition presents various advantages when compared to conventional filters; however, depending on the utilized polymer, their performance can still be insufficient to fully meet the growing demands of advanced filtration materials [20, 95]. Using polymers with intrinsic antimicrobial properties, such as CS, can enhance the overall efficiency of electrospun membranes as filtration devices, since they are capable not only of physically blocking particles but also of degrading or inactivating microorganisms present in the filtered air [96]. When polymers lacking such inherent properties are employed, the functionalization of nanofibers with bioactive compounds can introduce additional functionalities that further improve filtration performance. A variety of bioactive additives - including natural extracts (e.g., violacein, *Apocynum venetum* extracts, berberine hydrochloride, etc.), essential oils (e.g., cinnamon and peppermint, etc.), metal ions (e.g., silver, copper, etc.), metal oxide NPs (e.g., titanium dioxide, zinc oxide, etc.), and carbon-based materials (e.g., carbon nanotubes, fullerenes, and graphene) - have been shown to enhance the antimicrobial activity of electrospun membranes by killing or inactivating microorganisms on the facemask surface, thereby improving user safety [96–99]. Beyond antimicrobial enhancement, materials such as activated carbon, β-cyclodextrin, gelatin, and microporous polyimide metal–organic frameworks (MOFs) have been incorporated to increase the adsorption efficiency of VOCs [100, 101]. Furthermore, the addition of structural reinforcing agents such as cellulose nanocrystals (CNCs) and silica (SiO₂) NPs can improve the mechanical strength and durability of the electrospun membranes, contributing to the overall performance and longevity of air filters [100].

## Biodegradable polymers for air-filtrating applications

Polymers are characterized by their durability, strength and wide range of applications [102]. However, the resistance and degradation of certain polymers has become a serious problem for the environment. Non-biodegradable polymers, when improperly disposed of in the environment, persist in nature for decades [103]. Facemasks are generally single-use equipment and are mainly made of non-biodegradable polymers, as mentioned above. With the widespread use of masks, large quantities are being discarded, leading to environmental problems due to the release of large tons of microplastics into landfills and marine environments [104]. With this, biodegradable polymers, whose degradation is induced by bacteria and fungi through biological processes [105], have been an excellent alternative for a more sustainable society [106] by replacing non-biodegradable polymers.

Biodegradable polymers can be classified by their origin as natural or synthetic. Natural biodegradable polymers are abundantly present in nature, namely CS, gelatin, cellulose, among others. These polymers have several advantages such as biodegradability, biocompatibility and low toxicity [107, 108]. Therefore, natural polymers have been widely applied in areas of regenerative medicine, drug delivery systems, wound treatment, food packaging, etc. [58, 108–110]. On the other hand, synthetic biodegradable polymers, including PLA, PVA, PCL, PEG, among others, are materials produced with focus on physical–chemical and mechanical properties [111].

Biodegradable natural polymers and various proteins have been increasingly utilized in the development of bio-based materials, offering distinct advantages over synthetic polymers, particularly in terms of biodegradability and biocompatibility [112]. Additionally, they exhibit excellent filtration efficiency when electrospun into nanofibers, establishing them as appropriate bio-based air filtration alternatives to conventional filters manufactured from petroleum-based synthetic polymers. To enhance membrane performance, natural biopolymers have been combined with synthetic polymers [113]. A variety of strategies - including surface grafting, nanoparticle reinforcement, polymer blending, and the usage of bespoke copolymers - have been proposed and extensively investigated to further improve membrane properties. In this context, polymers such as PVA, polyvinylpyrrolidone (PVP), PEG, and PLA have been widely employed in the fabrication of polymeric membranes due to their high mechanical strength, biocompatibility, and recyclability [114].

Table 1 provides a general overview of the origin, composition, key characteristics, and functional properties of both natural and synthetic biodegradable polymers used for air filtration.

**Table 1:** Overview of natural and synthetic biodegradable polymers used for air filtering applications

Polymer		Origin and composition	Functional properties and characteristics	References
Natural polymers	Chitosan (CS)	Polysaccharide consisting of glucosamine and N-acetylglucosamine Obtained by N-acetylation of chitin	Biocompatible Biodegradable Antimicrobial, antioxidant and anti-inflammatory activities Non-toxic Good adsorption properties	[115–117]
	Gelatin	Protein produced by partial hydrolyzation of collagen	Biocompatible Biodegradable Highly hydrophilic	[118, 119]
	Cellulose acetate (CA)	Acetate ester of cellulose Obtained by acetylation of cellulose	Biocompatible Biodegradable Low toxicity Desired hydrophilic properties High chemical and mechanical stability	[120, 121]
Synthetic polymers	Poly(ε-caprolactone) (PCL)	Aliphatic polymer resulted from the polymerization of an open ring of ε-caprolactone or via free radical ring-opening polymerization of 2-methylene-1–3-dioxepane	Biodegradable Biocompatible Non-toxic Hydrophobic Highly permeable Bioresorbable Good mechanical properties	[122–124]
	Poly(lactic acid) (PLA)	Synthesized through polycondensation of lactic acid or ring-opening polymerization of lactide monomer	Biocompatible Biodegradable Hydrophobic Excellent mechanical properties	[125, 126]
	Polyethylene glycol (PEG)	Polyether created by the reaction of ethylene oxide with water, ethylene glycol or ethylene glycol oligomers Also known as polyethylene oxide (PEO) or polyoxyethylene (POE)	Biocompatible Biodegradable Non-toxic Hydrophilic Low immunogenicity	[127–129]
	Poly(vinyl alcohol) (PVA)	Copolymer composed of hydroxyl and acetyl units Produced by polymerization of vinyl esters or ethers (e.g., vinyl acetate), followed by saponification or transesterification	Biocompatible Biodegradable Non-toxic Hydrophilic Good thermal and chemical resistance	[130, 131]

## State of the art on biodegradable electrospun membranes with air-filtering and antimicrobial properties for facemask applications

Standard facemasks present several limitations, particularly from an environmental perspective. In contrast, biodegradable electrospun membranes offer significant potential as air-filtration media due to their favorable structural characteristics and sustainable nature. Nevertheless, challenges remain, including the survivability of microorganisms on facemask surfaces and the potential re-aerosolization of captured particles [132]. Therefore, the state-of-the-art research not only focuses on the use of biodegradable materials but also on enhancing their functional performance - particularly their ability to efficiently capture airborne particles and inactivate pathogens [12]. Recent years have seen an exponential rise in studies employing biopolymer-based nanofiber membranes for use in next-generation protective masks. Table 2 summarizes recent research efforts utilizing various biopolymers in the fabrication of electrospun air-filtration membranes exhibiting biodegradable and/or antimicrobial characteristics.

**Table 2:** Overview of recent studies on biodegradable air-filtering electrospun membranes with/without antibacterial properties

Membrane materials and characteristics	Average fiber diameter and pore size/porosity	Antimicrobial performance	Filtration performance	Pressure drop	Other results	References
PVA + soy protein isolate nanofibers	Average fiber diameter - 136 nm Average pore size - 4.4 μm	Not tested	99.90 – 99.99% for PM_10_ and 99.40 – 99.80% for PM_2.5_	80 to 350 Pa		[22]
Silk fibroin (SF) nanofibers	Average fiber diameter - 0.48 μm	Inhibition zones of *E. coli* and *S. aureus* were observed (obs.: for this test, SF nanofibers were doped with Ag NPs)	98.8% for 2.5 μm particles and 96.2% for 0.3 μm particles (tested with NaCl particles)	20 to 100 Pa	Low airflow resistance, light weight and biological compatibility	[133]
PVA + quaternary ammonium CS (HTCC) nanofibers	Fiber diameter range – 300 to 400 nm Pore size range - 110 to 160 nm	99.52% and 99.75% antibacterial rate was achieved with PVA-HTCC 6:4, for *E. coli* and *S. aureus*, respectively	82% for PM_1.0_, 86% for PM_2.5_ and 92% for PM_10_	Information not provided	Good thermal stability Uniform porous structure	[134]
PCL microfibers impregnated with Ag, TiO_2_ and MgO NPs	Average fiber diameter: PCL – 1.81 μm PCL + Ag NPs – 1.78 μm PCL + TiO2 NPs – 2.09 μm PCL + MgO NPs – 2.02 μm	Inhibition zones of 25.3 mm and 13.5 mm for *S. aureus* and *E. coli* respectively*,* achieved with the incorporation of MgO NPs	99.4% achieved with PCL and MgO NPs (tested with 1 µm polystyrene particles)	Information not provided	Good thermal comfort Decreased air permeability	[135]
PVA + Ag NPs nanofibers grafted with 3,3′,4,4′-benzophenone tetracarboxylic acid (BPTA)	Average fiber diameter – 220 nm	Inhibition zones with diameters of 18.12 ± 0.08 and 16.41 ± 0.05 mm against *S. aureus* and *E. coli*, respectively	99.98% (tested with 300 – 500 nm polydisperse NaCl particles)	168 Pa		[136]
Cellulose acetate (CA) + Cetylpyridinium bromide (CPB) nanofibers	Average fiber diameter – 0.2 μm Average pore size – 7.6 nm	Not tested	99.99% (tested with 7 – 300 nm NaCl particles)	300 to 2750 Pa		[137, 138]
CA + gum rosin nanofibers	Fiber diameter range – 327 to 430 nm Pore size < 1 μm	Average 93% bacterial growth inhibition against (tested against *S. aureus* and *K. pneumoniae*)	Highest value for filtration efficiency was 98.79% for CA + 15 g gum rosin nanofibers (tested with 5 × 10^5^ CFU/mL *S. aureus* inoculum)	Information not provided		[139]
Multilayer PVA + N-halamine biopolymer (P(ADMN-NVF)) + CS nanofibers	Fiber diameter range – 100 to 250 nm	Antibacterial assays suggest that chlorinated multilayer membranes have antibacterial ability against *E. coli* and *S. aureus*	99.3% for NaCl particles and 99.4% for DEHS particles (tested with 300 – 500 nm particles)	183 Pa for NaCl and 238 Pa for DEHS aerosols	Multilayer membrane composed with PVA/CS as outer and inner layers and PVA/P(ADMH-NVF) as intermediate layer	[140]
SF + PEO nanofibers	Average fiber diameter – 400 nm	Not tested	99.99% for PM_10_ and 99.84% for PM_2.5_ (tested using smoke particles)	75 Pa		[141]
PLA nanofibers	Fiber diameter range – 273.6 to 721.5 nm Porosity range - 75.3 to 87.1%	Not tested	Lowest filtration efficiency was 99.7% (tested with 260 nm average diameter NaCl particles)	165.3 Pa		[142]
PLA + TiO_2_ NPs nanofibers	Fiber diameter range – 1.31 to 1.40 μm (with increased concentration of TiO_2_ NPs) Pore size range – 10 to 120 nm	Removal efficiency of 99.5% against *S. aureus*	Lowest filtration efficiency was 99.965% (tested with 260 nm NaCl particles)	128.7 Pa		[143]
PVA + cellulose nanocrystals (CNC) nanofibers	Fiber diameter range – 127.6 to 209.4 nm	Not tested	99.1% for PM_2.5_ (tested with < 0.3 to > 10 μm burned incense particles)	91 Pa		[144]
PVA + AgHEC NPs nanofibers	Average fiber diameter – 434 nm	Reductions of 99.6% against *S. aureus* and 100% against *E. coli*	99.6% and 97.5% for air velocity of 5.5 cm s^−1^ and 16.7 cm s^−1^, respectively (tested with 1 μm polystyrene latex and 10 – 700 nm NaCl and particles, for a 5-layer structure of PVA/AgHEC nanofibers)	59 Pa	Optimal efficiency/breathability balance Very good stability, even after 24 h of water contact	[145]
CS + PCL nanofibers	Fiber diameter range – 200 to 400 nm	Significant reduction *S. aureus* adhesion with CS-PCL membranes compared with pure PCL membranes	99.76% for 300 nm particles and 100% for 1000 nm particles with 25%CS/75%PCL membrane (tested with 100, 300 and 1000 nm polystyrene particles)	Information not provided		[146]
PVA/PAA/Ag/SiO_2_ NPs nanofibers	Fiber diameter range – 0.2 to 0.8 μm	Antibacterial activities against *E. coli* and *Bacillus subtilis (B. subtilis)*	98% (tested with 300 – 500 nm NaCl and diisooctyl sebacate (DEHS) particles)	Information not provided	High tensile strength, biological compatibility, low airflow resistance	[147]
PEO + zein nanofibers	Fiber diameter range – 200 to 440 nm Pore size range – 2 to 4 μm	Not tested	> 99.6% for membranes with zein (tested with 0.3 - 10 μm particles)	100 to 250 Pa	Zein-based membranes demonstrated excellent moisture resistance, due to their inherent hydrophobic nature	[148]
PLA + CS NPs nanofibers	Fiber diameter range – 1.21 to 1.35 μm Pore size range – 4.50 to 8 μm	99.5% and 99.4% reduction of *S. aureus* and *E. coli*, respectively	98.10 - 98.99% using different concentrations of CS NPs (tested with 0.26 µm sodium chloride (NaCl) particles)	147.60 Pa	The membranes containing more CS exhibited better antibacterial performance	[149]
Deposition of a nitrogen-doped TiO_2_ (N-TiO_2_) and TiO_2_ mixture in PVA + PEO + CNF nanofibers	Average fiber diameter – 790 nm Pore size range – 2 to 3 μm	Under light radiation, 100% sterilization of bacteria (*E. coli* and *S. aureus*) was achieved (although most bacteria were found viable without light radiation)	98.7% (tested with 75 nm NaCl particles)	Information not provided	The used mask can be rejuvenated through light irradiation and reused Excellent cycling performance, wearability, and stable filtration efficiency after 120 min wearing Excellent breathability (83.9 L/min)	[150]
CS + PVA + Ag/TiO_2_ NPs nanofibers	Fiber diameter range – 25.6 to 92.8 nm	Mortality rate of 97% for *E. coli* and 99% for *S. aureus* within 2 h (for nanofibers with Ag NPs) and 90% for *E. coli* and 92% for *S. aureus* (for nanofibers with TiO_2_ NPs)	> 99% (tested with 75 nm monodisperse NaCl particles)	20 to 85 mm H_2_O	Excellent water durability and stable microstructure in high humidity and water immersion	[151]
PVA + benzalkonium chloride (BAC) nanofibers	Fiber diameter range – 91 to 701 nm	Significant reduction in colony-forming units (CFU) after two hours of contact with *E. coli* and *S. aureus* Viral reduction of 99% against human coronavirus HCoV-229E	85.84% for 0.3 μm particles, 98.37% for 0.5 μm particles and 99.79% for 3 μm particles	26 to 256 Pa	PVA-based nanofibers were collected on a melt blown polypropylene nonwoven textile, due to their low mechanical properties	[152]
PLA + CuMOF nanofibers	Fiber diameter range – 400 to 700 nm	Inhibition zone of 3.0 cm (in the dark) and 3.3 cm (with light) for *S. aureus*	89.4% for 0.3 μm particles and 93.97% for 0.5 μm particles (tested with 0.225 – 12.5 μm NaCl particles)	20 to 100 Pa	Robust self-cleaning capability and satisfactory thermal comfort	[153]
PCL + zein + Ag NPs nanofibers	Porosity range - 80.53 to 87.6% (lowest pore size – 922 nm)	Removal efficiencies of 98.1% and 97.4% for *E. coli* and *S. aureus*, respectively 97.1% virus removal efficiency after 60 min with *E. coli* phage	97.19 – 99.19% (tested with 300 – 1000 nm NaCl particles)	72 to 510 Pa	Biodegradability was evaluated, with these membranes presenting considerable weight and size loss, and became viscous and soft (after nine days in the soil)	[154]
PVA + KGM + ZnO NPs nanofibers	Fiber diameter range – 0.273 to 0.398 μm Pore size range – 0.6 to 25 nm	Inhibition zones of 14 to 16 mm and 12 to 15 mm for *B. subtilis* and *E. coli*, respectively, using different concentrations of ZnO	99.99% for 1.0 wt% of ZnO (tested with 0.3 µm NaCl particles)	89 to 158 Pa	Incorporation of ZnO NPs resulted in superior photocatalytic activity	[155]
PLA + zein nanofibers	Fiber diameter range – 0.15 to 0.35 μm	Not tested	98.14% for PM_2.5_ and 97.39% for PM_10_ using a 1:1 PLA:zein ration (tested with oily and aqueous particles)	5 to 7.5 mbar	Evaluation of biodegradability show an almost complete degradation of PLA-zein membranes after 21 days Membranes also showed a great filtration efficiency for both aqueous and oily particles	[156]

**Fig. 4:**
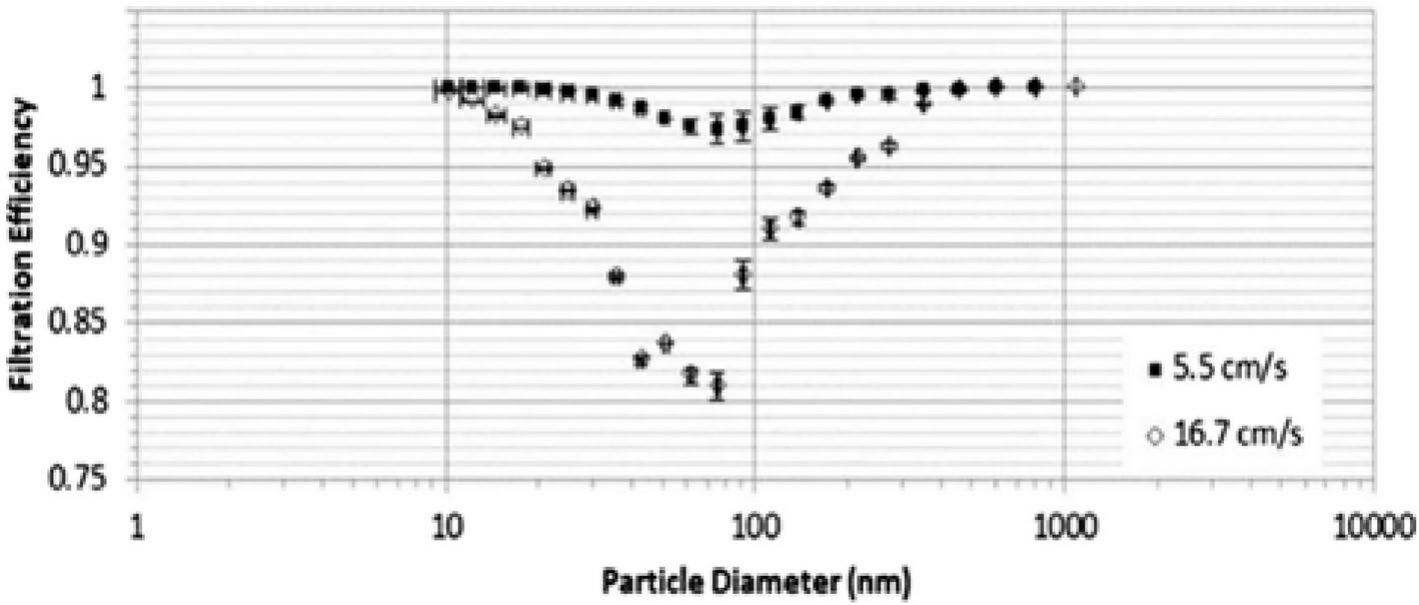
Filtration efficiency of electrospun PVA/AgHEC nanoparticle membranes against NaCl aerosols of varying particle sizes. Retrieved from [145]

Filtration efficiency is a critical performance indicator for biodegradable electrospun membranes used in air filtration, as it reflects their ability to effectively capture airborne particles. These membranes consistently exhibit high filtration efficiency across a broad range of particle sizes while maintaining good breathability, a feature that is characterized by high porosity (typically exceeding 80%) and a low pressure drop (generally below 200 Pa) [133–140]. These attributes emphasize their potential as sustainable alternatives to the conventional filter layers used in surgical masks. For instance, silk fibroin (SF) blended with PEO achieved a filtration efficiency of 99.99% for PM_10_ and 99.84% for PM_2.5_, with a pressure drop as low as 75 Pa, resulting in good breathability [141]. Similarly, PLA nanofibers exhibited maximum porosity of 87% and a pressure drop of 165.3 Pa, while maintaining excellent filtration performance, with the lowest reported filtration efficiency reaching 99.7% [142]. The incorporation of active components into electrospun membranes has been shown not to compromise their filtration performance. For instance, PLA nanofibers functionalized with TiO_2_ NPs demonstrated pore sizes ranging from 10 to 120 nm, a low pressure drop (128.7 Pa), and a high filtration efficiency of 99.965% [143]. Similarly, the addition of cellulose nanocrystals to PVA-based electrospun membranes improved the overall filtration efficiency of the membranes, reaching 99.1% with a pressure drop of only 91 Pa [144]. Moreover, PVA membranes functionalized with AgHEC NPs exhibited filtration efficiencies of 99.6% and 97.5% at particle velocities of 5.5 cm·s⁻^1^ and 16.7 cm·s⁻^1^, respectively (Fig. 4), demonstrating that electrospun membranes can maintain high filtration performance even under increased airflow velocities [145]. While these findings demonstrate that biodegradable polymer-based electrospun membranes can achieve high filtration efficiency under controlled laboratory conditions, further studies evaluating these membranes under more realistic conditions are necessary to fully assess their practical applicability. Nonetheless, the overall results indicate that such materials are a promising approach for air filtration applications, offering a balance between high filtration efficiency and favorable breathability.

Another key aspect in the development of sustainable electrospun filters is the incorporation of active antimicrobial functionalities, designed to inactivate microorganisms present on the surface of the facemask, thereby preventing re-aerosolization and reducing the risk of secondary infection. Some biopolymer blends inherently possess these antimicrobial properties, showing growth inhibition of Gram-positive and Gram-negative bacteria. For instance, the incorporation of CS into PCL membranes resulted in a significant reduction in *Staphylococcus aureus (S. aureus)* adhesion compared to pure PCL membranes, while maintaining near-100% filtration efficiency [146].

**Fig. 5:**
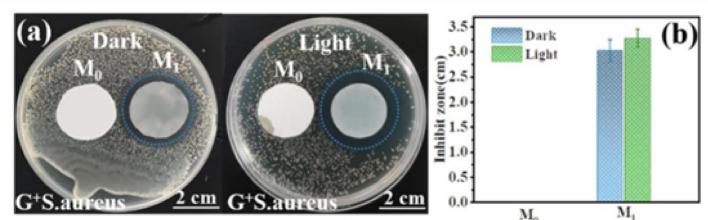
Antibacterial assessment of PLA (M0) and PLA–CuMOF (M1) electrospun membranes against *S. aureus*: **a** inhibition zones under dark and light conditions; **b** comparison of the average inhibition zone diameters observed under both conditions. Retrieved from [153]

Furthermore, electrospun membranes can be functionalized with a diverse range of antimicrobial and adsorptive agents, such as metal and metal oxide NPs (Ag, Cu, MgO, ZnO, TiO₂), MOFs, and activated carbon, showing antimicrobial activity without compromising filtration efficiency [133, 135, 136, 147–149]. For example, the deposition of TiO₂ and nitrogen-doped TiO₂ NPs onto PVA–PEO–cellulose nanofiber (CNF) membranes achieved 100% sterilization against *Escherichia coli (E. coli)* and *S. aureus* under light irradiation, achieving 98.7% filtration efficiency [150]. Similarly, PVA–CS membranes functionalized with Ag NPs exhibited mortality rates of 97% against *E. coli* and 99% against *S. aureus* within 2 h, while ensuring filtration efficiencies exceeding 99% [151]. PVA nanofibers functionalized with benzalkonium chloride (BAC) exhibited a significant reduction in colony-forming units (CFUs) after 2 h of contact with *E. coli* and *S. aureus*, as well as a 99% reduction in a human coronavirus cell line (HCoV-229E). Notably, these membranes maintained excellent filtration performance, achieving efficiencies exceeding 98% for 0.5 µm particles and approaching 100% for 3 µm particles [152]. PLA membranes functionalized with CuMOFs exhibited pronounced antibacterial activity (Fig. 5), generating inhibition zones of 3.0 cm under dark conditions and 3.3 cm upon light exposure. In addition, these membranes achieved a filtration efficiency of approximately 94% for particles with a diameter of 0.5 µm [153]. Therefore, the development of electrospun membranes with multifunctional bioactivity opens new pathways for promoting user health, making them highly promising for next-generation facemask technologies.

The focus of this research area is to address the limitations of conventional facemasks, namely their single-use design and improper disposal, which contribute to microplastic accumulation and environmental pollution. To this end, the development of filtering membranes prioritizing biodegradability and reusability has become a key objective. Several studies have reported electrospun membranes with these capabilities, demonstrating self-cleaning properties and/or the ability to be reused [137, 145]. For instance, PCL–zein membranes containing Ag NPs were submitted to biodegradability tests, showing weight loss, increased softness, and a viscous appearance after nine days of soil burial [154]. Furthermore, PVA-based membranes incorporated with konjac glucomannan (KGM) and ZnO NPs exhibited enhanced photocatalytic activity [155], while PVA-PEO-CNF membranes doped with N-TiO₂/TiO₂ NPs maintained antibacterial activity and filtration efficiency after multiple cycles of light-based rejuvenation, sterilization, and washing [150]. PLA–zein electrospun membranes also exhibited high filtration efficiency, while maintaining the ability to undergo significant biodegradation after 21 days of soil burial, highlighting their potential as sustainable filtration materials [156]. Currently, the lack of standardized protocols for evaluating the biodegradability of polymer-based nanofibrous filters remains a challenge, particularly with respect to testing under conditions that accurately reflect real-world disposal environments. Existing methods vary significantly depending on the methodology and environmental parameters employed, which limits the comparability of results. Therefore, future research should focus on the development of standardized and reproducible biodegradability testing protocols that account for relevant environmental factors (e.g., temperature, humidity, microbial activity), in order to more reliably assess the environmental performance and sustainability of biodegradable nanofibrous materials. Nevertheless, the present findings highlight the potential of electrospun membranes for sustainable, multifunctional facemask applications, contributing to a circular approach in PPE design.

**Fig. 6:**
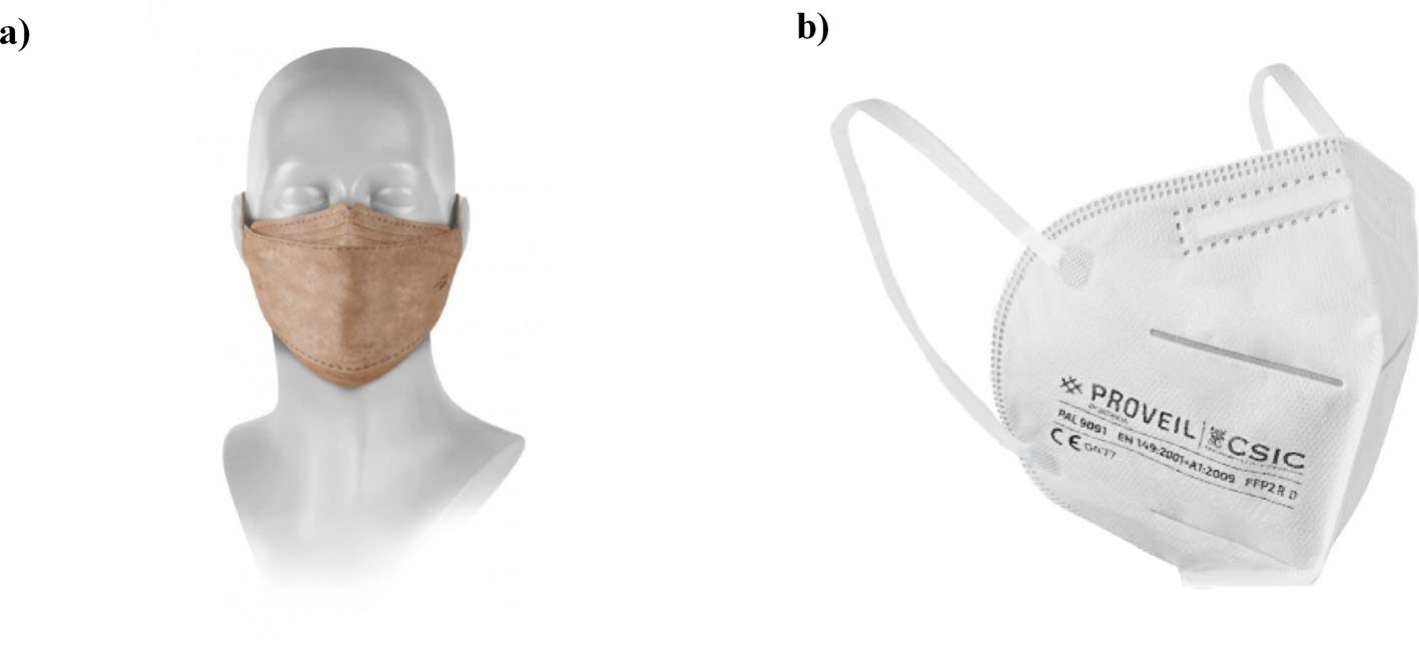
**a** Nanofiber-based self-sterilizing FFP2 NR respirator developed by Respilon [157]; (b) FFP2 facemask based on nanofibrous technology developed by Bioinicia in collaboration with CSIC, commercially available through Proveil [160]

To develop alternatives to conventional surgical facemasks, several companies have focused on the industrial-scale fabrication of electrospun nanofibers and the design of protective facemasks incorporating filtration layers composed of nanofiber materials. Although major advances have been achieved in the production of nanofiber-based protective equipment (illustrated in Fig. 6) by companies such as Respilon [157], Kymira [158] and NASK [159], which already offer commercially available products, fully biodegradable nanofiber-based facemasks remain limited in both development and market availability. Among these, Bioinicia®, in collaboration with the Spanish National Research Council (CSIC), developed the Bio Hygienic Mask, which exhibits FFP2-level filtration efficiency (up to 98% aerosol retention) and biodegrades within 22 days upon exposure to water and CO_2_ [160]. Similarly, the startup ÖKOSIX has introduced a nanotechnology-based surgical mask composed of biodegradable materials, capable of degrading within six months, while maintaining medical-grade ASTM Level 3 filtration performance [161, 162]. Lepton/NanofiberLabs has also proposed the development of a fully PLA-based facemask, utilizing PLA nonwoven fabric for both the outer and inner layers, while the filter layer is designed to consist of PLA nonwoven fabric combined with electrospun PLA nanofibers, and the ear loops fabricated from PLA material [163]. However, it should be noted that biodegradability claims for these commercial products are often based on specific testing conditions, and evaluation under standard environmental conditions is limited. In some cases, degradation may require controlled environments, which are not representative of typical disposal scenarios. Therefore, these claims should be interpreted with caution.

## Natural fibers as an alternative for inner and outer layers of facemasks

**Fig. 7:**
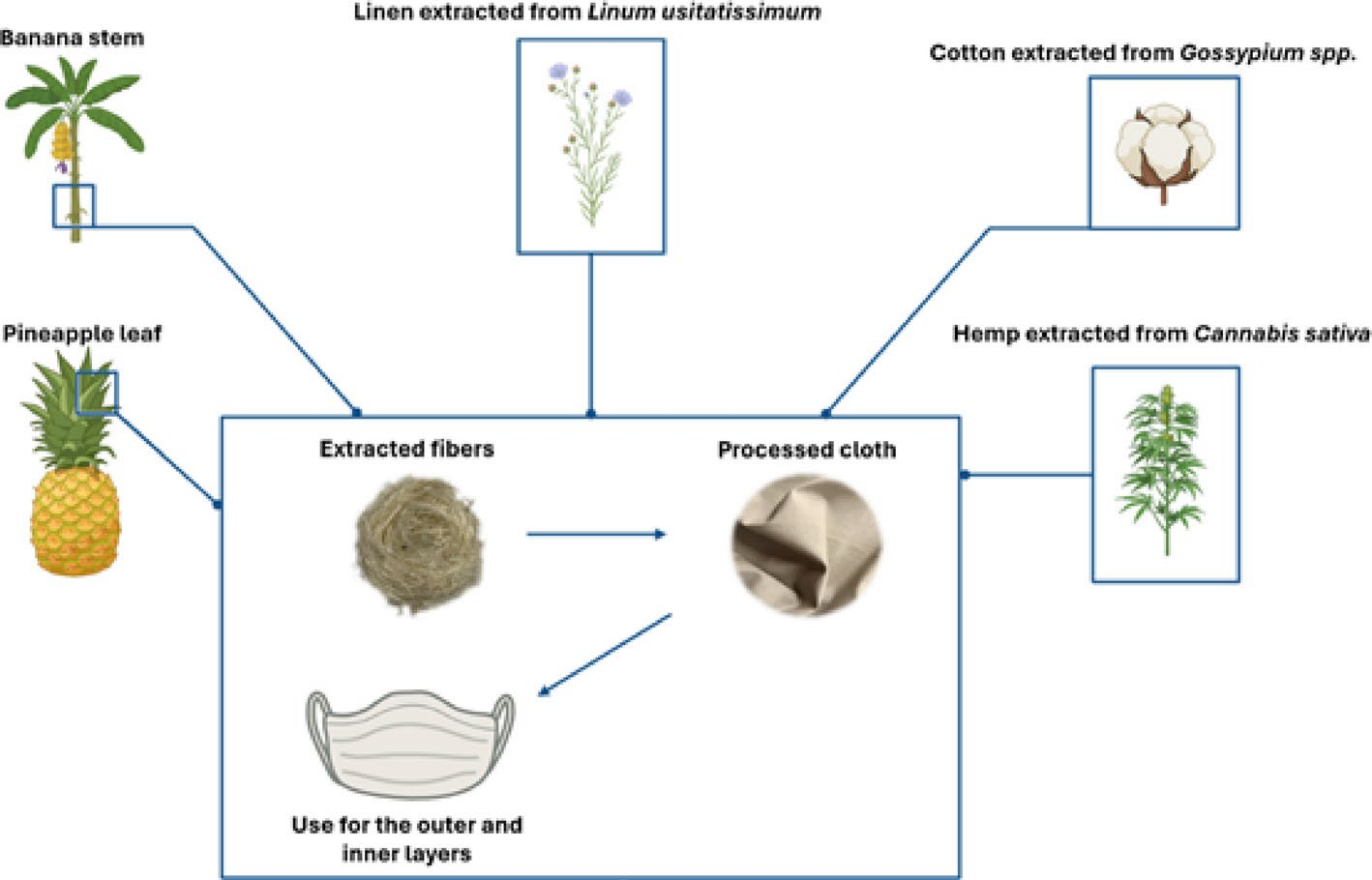
Schematic representation of natural fibers—banana stem fiber, pineapple leaf fiber, linen obtained from *Linum usitatissimum*, cotton obtained from *Gossypium* spp., and hemp derived from *Cannabis sativa*—utilized in the fabrication of the outer and inner layers of a fully biodegradable facemask

The excessive disposal of non-degradable facemasks after short periods of use has prompted research into natural materials for the development of novel facemasks, aiming not only to effectively filter pathogens but also to serve as a more sustainable alternative. Natural cellulosic fibers (cotton, flax, hemp, etc.) possess several favorable characteristics for this purpose, including good mechanical strength, hydrophilicity, and biocompatibility [11]. Such fibers also present good breathability and are comfortable in terms of feel and smell [164], making them suitable candidates for the outer and inner layers of a facemask (as described in Fig. 7). According to Gbadegbe et al*.*, cotton is recommended as the outer layer in multilayered fabrics due to its advantageous properties, including high air permeability, user comfort, and smooth texture. The inner fabric, however, is recommended to be made from linen or silk in order to provide a smooth and soft feel against the wearer’s skin [164].

Cotton is the most popular consumable natural fiber because of its many advantageous features, including natural look, heat transfer, wearing comfort, moisture absorption, and renewable status. Cotton fibers have tensile strengths ranging from 287 to 800 MPa and a Young's modulus ranging from 5.50 to 13.0 GPa [165]. Cotton fibers are mostly utilized for clothing, and because clothes come into touch with external microbes, they can act as microorganism carriers. New antibacterial cotton fibers are being explored as a way to incorporate antimicrobial characteristics into garment materials, proving to be beneficial as a prevention mechanism for skin allergies. In vitro studies on functionalized cotton fibers with NPs against bacteria revealed that the polar fiber and cationic ions from these particles interact with the anionic features of the bacteria cell envelope, causing denaturation of several proteins of bacterial membranes and resulting in the bacteriolytic effect in Gram-positive and Gram-negative bacteria. Another proven coating for cotton fibers is antimicrobial protein functionalization to promote microbial growth suppression with effective results against Gram-positive bacteria *B. subtilis* and Gram-negative bacteria *E. coli* assessed by Xu et al. Depending on the bacteria, cotton fibers had good to outstanding antibacterial activity [166, 167]. Ho et al. [168] reported that a self-designed three-layered cellulosic cotton mask should be considered a potential substitute for medical masks, which are currently made of synthetic polymer.

Linen fibers have the advantages of low-cost production, low density, and biodegradability, being used in the production of textiles, composites and papers. These fibers provide various benefits, including air permeability and comfort in clothes [169, 170]. Xiao et al [171] tested the filtration efficiency of linen under sneeze-like pressure. The results showed that 2 layers of linen could block 53.2% of the microspheres and 80.3% of the starch particles, suggesting that it has the ability to block some microdroplets.

Hemp is another natural fiber with a significant surface area, possessing several key mechanical characteristics including high tensile strength (550–1110 MPa) and Young's modulus (30–70 GPa) [172]. Additionally, hemp has proven to be effective in the inhibition of the growth of different types of bacteria, making them suitable for this type of application [173, 174].

New non-conventional natural fibers such as banana stem fiber and pineapple leaf fiber have the potential to be utilized in the development of biodegradable facemasks [175, 176]. Aguilar et al. [177] assessed the air permeability and water repellency of a banana pseudo-stem fiber facemask. The results showed that the facemask has high air permeability and water repellency. Wiryadinata et al. [178] produced a facemask using pineapple leaf fiber with advantages in texture, weight, thickness, tensile strength, wet pull resistance and good ply bond.

Exploring natural fibers for facemasks production offers a promising solution to the environmental concerns posed by non-degradable masks, particularly for the inner and outer layers. Natural materials such as cotton, silk, linen, hemp and new alternatives like banana and pineapple leaf fibers demonstrate desirable properties (breathability, mechanical strength, antimicrobial activity and biodegradability) to support effective filtration and contribute to comfort and sustainability, making them viable candidates for eco-friendly facemasks.

## Conclusions

The increasing concern over air quality and environmental sustainability has driven the search for advanced filtration materials that not only ensure effective protection against airborne particulates and pathogens but also minimize ecological impact. Conventional facemasks contribute substantially to environmental pollution due to their resistance to degradation and widespread single-use design. In contrast, biodegradable polymers and natural fibers represent a viable and sustainable pathway toward next-generation filtration systems that combine high performance with environmental responsibility.

Electrospun nanofiber membranes have demonstrated exceptional potential for facemask applications owing to their intrinsic advantages. When fabricated from biodegradable polymers, these membranes can deliver high filtration efficiency, low pressure drop, and effective microbial inactivation, without compromising breathability or wearer comfort. However, industrial-scale translation can present challenges, primarily due to limitations in mass production processes and the need for precise parameter control, which may lead to fiber morphology inconsistencies. One of the main issues associated with electrospinning scale-up is the use of increased flow rates, which can result in defect formation and morphological changes, such as larger pore sizes and increased fiber diameters, affecting key structural properties, including filtration efficiency. Although these challenges remain significant, several strategies have been proposed to mitigate them, including multi-needle systems, free-surface electrospinning techniques, optimized collector and spinneret designs, and the application of auxiliary forces to improve process stability and fiber uniformity [179].

The incorporation of natural fibers in the outer and inner layers further enhances user comfort, promotes biodegradability, and reduces reliance on synthetic plastics. Importantly, while model air samples provide a standard for performance evaluation, it is essential to test electrospun membranes under real-world conditions to accurately evaluate their effectiveness in practical air filtration scenarios. Ultimately, the integration of biodegradable electrospun membranes with natural fiber layers offers a reliable and environmentally responsible solution to ease the environmental burden of disposable facemasks, while maintaining high standards of filtration efficiency, antimicrobial protection, and user comfort.

## Data Availability

No datasets were generated or analysed during the current study.
